# Adaptation of *Staphylococcus aureus* to the Human Skin Environment Identified Using an *ex vivo* Tissue Model

**DOI:** 10.3389/fmicb.2021.728989

**Published:** 2021-09-21

**Authors:** Marc Burian, Johanna Plange, Laurenz Schmitt, Anke Kaschke, Yvonne Marquardt, Laura Huth, Jens M. Baron, Mathias W. Hornef, Christiane Wolz, Amir S. Yazdi

**Affiliations:** ^1^Department of Dermatology and Allergology, RWTH University Hospital Aachen, Aachen, Germany; ^2^Institute of Medical Microbiology, RWTH University Hospital Aachen, Aachen, Germany; ^3^Interfaculty Institute of Microbiology and Infection Medicine, University of Tuebingen, Tuebingen, Germany

**Keywords:** *ex vivo* skin explant, proteases, global regulators, accessory gene regulator (*agr*), bacterial adhesion, immune evasion, colonization, human skin (*in vivo*)

## Abstract

The healthy human epidermis provides physical protection and is impenetrable for pathogenic microbes. Nevertheless, commensal and pathogen bacteria such as *Staphylococcus aureus* are able to colonize the skin surface, which may subsequently lead to infection. To identify and characterize regulatory elements facilitating adaptation of *S. aureus* to the human skin environment we used *ex vivo* tissue explants and quantified *S. aureus* gene transcription during co-culture. This analysis provided evidence for a significant downregulation of the global virulence regulator *agr* upon initial contact with skin, regardless of the growth phase of *S. aureus* prior to co-culture. In contrast, the alternative sigma factor B (*sig*B) and the antimicrobial peptide-sensing system (*gra*RS) were expressed during early colonization. Consistently, *sig*B target genes such as the clumping factor A (*clf*A) and fibrinogen and fibronectin binding protein A (*fnb*A) were strongly upregulated upon skin contact. At later timepoints of the adhesion process, wall teichoic acid (WTA) synthesis was induced. Besides the expression of adhesive molecules, transcription of molecules involved in immune evasion were increased during late colonization (staphylococcal complement inhibitor and staphylokinase). Similar to nasal colonization, enzymes involved in cell wall metabolism (*sce*D and *atl*A) were highly transcribed. Finally, we detected a strong expression of proteases from all three catalytic classes during the entire colonization process. Taken together, we here present an *ex vivo* skin colonization model that allows the detailed characterization of the bacterial adaptation to the skin environment.

## Introduction

Healthy skin protects from microbial colonization and infection through a variety of mechanisms such as a competitive microbiota and a large number of antimicrobial and immunological effector molecules including lipids, salts, enzymes, and an acidic surface pH (Chen et al., 2018). Under diseased conditions, promoted by changes in the microbiota composition, deficiency of skin barrier components and loss of skin barrier integrity, pathogens such as *Staphylococcus aureus* can cause severe skin and soft tissue infections (Olaniyi et al., 2017). Besides these infections, *S. aureus* colonizes lesional skin of atopic dermatitis (AD) patients especially during an eczema flare. Here the diversity of the normal microflora is drastically reduced and the increased abundance of *S. aureus* is linked to the severity and worsening of this chronic inflammatory skin disease (Totte et al., 2016). Beside its primary ecological niche—the human nose—the skin represents a second important reservoir of *S. aureus* in AD patients (Alsterholm et al., 2017). In contrast to atopic skin, only ∼10–20% of healthy subjects are colonized with *S. aureus* on the skin (Matsui et al., 2000; Shi et al., 2016). Only limited information is available on *S. aureus* factors required to colonize the healthy skin and the concomitant expression of putative virulence factors. It is likely that some of these factors are induced upon contact of *S. aureus* with the skin surface or by specific skin micronutrients or environmental factors. *S. aureus* is equipped with several interactive regulatory systems, which orchestrate appropriate gene expression during colonization and infection (Jenul and Horswill, 2019). The information on direct *ex vivo* or *in vivo* bacterial gene expression is so far limited. We recently characterized *S. aureus* gene expression during persistent nose colonization of healthy volunteers (Burian et al., 2010a,b, 2012) and during the initial phase of nasal colonization in cotton rats (Burian et al., 2010a). From these analyses it became evident that the global virulence regulators (*agr*, *sae*, *sig*B, *gra*RS) were not activated during colonization, whereas expression of the essential two-component regulatory system *wal*KR was increased compared to *in vitro* grown bacteria. Furthermore, nasal *S. aureus* colonization was characterized by the expression of genes mediating adhesion (*clf*B, *fnb*A, *isd*A, *tag*O), cell surface dynamics/remodeling (*sce*D, *atl*A, *oat*A), the expression of immune evasion genes (*sak*, *chp*) and the lack of toxin transcription (*hla psm*). We hypothesize that gene expression might differ significantly once *S. aureus* leaves its habitat in the nose and adapts to the skin environment and that such regulatory switches contribute to successful colonization. On the long-term, a better understanding of the specific gene expression pattern will give insight in the importance of single highly expressed factors and the importance of regulatory systems.

In the present study, we established an *ex vivo* skin explants model to investigate *S. aureus* adaption to human skin. The model is similar to a recent described model for the investigation of topical therapeutics (Neil et al., 2020). Our model allows continuous monitoring of gene expression changes up to 8 days after initial contact with *S. aureus*. Here, the colonization of healthy skin led to a uniform response of the pathogen independently of the human host and a similar gene expression pattern was observed when *S. aureus* from exponential or post exponential growth phase were inoculated. In this model which is largely deprived of immune cells, the effects of *S. aureus* adaptation by direct contact to the human skin can be determined, independently of infiltrating immune cells.

## Materials and Methods

### Bacterial Strains and Growth Conditions

*S. aureus* USA300 JE2 (Fey et al., 2013) was grown overnight in CnT-Prime medium (CELLnTEC), diluted to an initial optical density value at 600 nm (OD_600_) of 0.05 in fresh CnT-Prime medium and grown with shaking (180 rpm) at 37°C either to the exponential (OD_600_ = 0.5) or post-exponential (OD_600_ = 0.5 + 4 h) growth phase.

### Ethics Statement

*Ex vivo* skin explants were obtained from skin tumor surgery in which pieces of healthy skin were removed for surgical reasons to compensate for the circular excisions upon surgical closure. This approach was approved by the local ethics committee of the Medical Faculty RWTH University of Aachen, Germany (EK 404/19). In accordance with the Declaration of Helsinki Principles guidelines written consent was obtained from all participants involved in the study.

### Human *ex vivo* Skin Model

Immediately after surgery, subcutaneous adipose tissue was removed and skin explants were punched into defined pieces (4 mm in diameter) using surgical biopsy punches (pfm medical AG). Skin samples were incubated for 12 h in DMEM (Pan Biotech) supplemented with 10% fetal calf serum (Pan Biotech) and 1x antibiotic-antimycotic (Gibco^TM^ 15240062) to remove the remaining skin flora. The skin area was routinely disinfected prior to surgery and subsequent incubation in DMEM supplemented with 1x antibiotic-antimycotic (12 h) ensured a significant reduction of the natural skin flora. In order to enable colonization with *S. aureus* samples were washed twice with pre-warmed 37°C phosphate-buffered saline (PBS) [Pan Biotech] and placed into Millicell^®^ 24-well cell culture inserts with a 0.4μm pore size membrane (Merck). Inserts were placed into 24-well plates prefilled with 300 μl of CnT-Prime medium and explants were cultured at the air-liquid interface at 37°C and 5% CO_2_. After 4 days, CnT-Prime medium was added to the wells, ensuring the continuous supply of nutrients to the tissue from below. Either exponential or post-exponential grown *S. aureus* USA300 JE2 bacteria were harvested by centrifugation at 4.000 rpm for 10 min and resuspended in CnT-Prime medium. 9 μl of bacterial suspension corresponding to 1 × 10^6^ colony-forming units (CFUs) were applied step wise in 3–4 μl steps to the surface of the skin explants.

As a control, we randomly applied the concentrated medium to blood agar plates to examine for the presence of *S. aureus*. No growth of *S. aureus* was detected in any of the plates (data not shown). For transcript analyses infected skin samples were incubated for 15 min, 1, 2, 4, 6, 18, 28, and 74 h, as well as 5, 6, 7, and 8 days at 37°C and 5% CO_2_. At the defined times, skin explants were removed from the inserts, cut into small pieces and dissolved in 1 ml TRIzol^TM^ LS reagent (Thermo Fisher Scientific) for RNA isolation. The bacterial inoculum that was applied to the skin’s surface was also transferred into 1 ml of TRIzol^TM^ LS reagent. In addition, wells containing 300 μl of CnT Prime medium only, were also inoculated with 1 × 10^6^ CFUs of the inoculum suspension to analyze the *in vitro* growth without skin. Bacteria grown in medium only were also transferred into 1 ml of TRIzol^TM^ LS reagent for RNA isolation at the same time points already defined for the *ex vivo* skin explants up to 18 h.

### RNA Isolation, Reverse Transcription and Quantitative Real-Time PCR

Bacteria were lysed and RNA isolation was performed as described previously (Burian et al., 2010a). To eliminate contaminating DNA each RNA sample was digested with 8 U of RNase-free DNase I (Roche), 2 μl 10 × incubation buffer (Roche), and 16 U of RNasin ribonuclease inhibitor (Promega) for 30 min at 25°C. DNase treatment was carried out twice for each sample. DNase I treatment was stopped using DNase inactivation reagent (Thermo Fisher Scientific). Three microliter of total RNA were transcribed into cDNA using Super Script III Reverse Transcriptase (Thermo Fisher Scientific) and 200 ng of random hexamer primers (Thermo Fisher Scientific). Reverse transcription was performed as described in the instructions of the Superscript manufacturer. cDNAs were diluted 1:3 with nuclease-free water (Thermo Fisher Scientific) and frozen at −20°C using Eppendorf LoBind Tubes (Eppendorf, Hamburg, Germany) for prolonged storage.

Relative quantification of *S. aureus* transcripts by qPCR was carried out using the 7300 Real Time PCR instrument (Applied Biosystems) in combination with the KAPA SYBR^®^ FAST qPCR Master Mix (2x) ABI Prism (Merck). Master mixes were prepared as following: 8 μl KAPA SYBR^®^ FAST qPCR Master Mix (2x) ABI Prism, 8 μl nuclease-free water, 1 μl of each primer (see Supplementary Table 1; Burian et al., 2010a,b), and 2 μl cDNA. The following temperature profile was utilized for amplification: denaturation for 1 cycle at 95°C for 15 s and 55 cycles at 95°C for 27s, 55–60°C for 10 s, and 72°C for 27 s with fluorescence acquisition at 72°C. Melting-curve analysis was done at 60–97°C with stepwise fluorescence acquisition. Relative quantities of transcripts were calculated by a standard curve for each gene generated using sixfold serial dilution of *S. aureus* USA300 wild type RNA.

### Skin Explant Viability by Membrane-Permeable Dye Thiazolyl Blue Tetrazolium Bromide Assay

The membrane-permeable dye thiazolyl blue tetrazolium bromide (MTT) was used to assess skin explant viability. Four millimeter skin biopsies were cultured as described above up to 10 days. From day 5 onward skin explants were washed twice with PBS and further incubated in 2 mg/ml MTT (Sigma-Aldrich) for 2 h at 37°C and 5% CO_2_. MTT is reduced from yellow color to purple formazan in living skin biopsies.

### TUNEL Assay

Apoptosis was assessed on skin tissue using an *in situ* Apoptosis Detection Kit (Sigma-Aldrich) according to the manufacturer’s protocol. After the final washing step, sections were mounted in ibidi Mounting Medium with DAPI (ibidi). Staining was visualized using a Leica DMI4000 B microscope (Leica).

### Histological Analysis and Immunostaining

Hematoxylin and eosin (H&E) staining, as well as immunostaining, were carried out as previously described by Huth et al. (2018). Briefly, for immunofluorescence, 4 μm cryosections were fixed for 10 min in acetone at 4°C. First antibodies keratin 10 (Dako) and filaggrin (AKH1) (Santa Cruz) were diluted with Antibody Diluent (Dako) and incubated at room temperature for 1 h. Following washing steps with PBS, the sections were incubated in fluorochrome-conjugated secondary antibody Alexa Fluor 488 IgG H + L (Molecular Probes) for 1 h at room temperature. Cell nuclei were stained with DAPI (Applichem). All image processing and analyses were performed with ImageJ software (National Institutes of Health).

### Statistical Analysis

Statistical analysis was performed with the Prism 9.0.2 package (GraphPad Software) using multiple *t*-tests. *P* < 0.05 was considered to be statistically significant.

## Results

### Viability of Skin Explants

**FIGURE 1:**
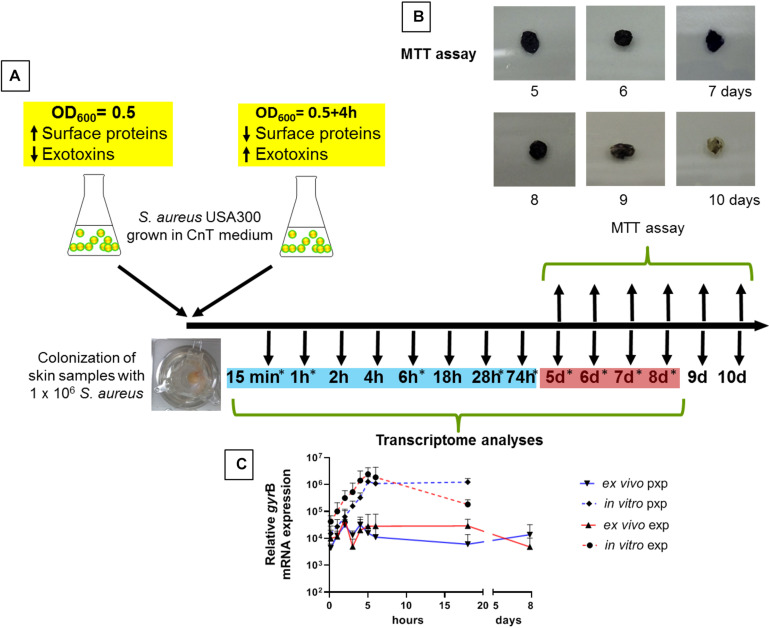
Skin colonization model and experimental time course. **(A)** To analyze the adaptation of *S. aureus* to the epidermis, skin explants were colonized with either exponentially or post-exponentially grown *S. aureus* USA300 and transcript analyzes were performed up to 18 h. For a broad screening of genes, skin samples were colonized with exponentially grown bacteria and transcript analyses were carried out at 9 different times indicated with an asterisk. Time points marked in blue are defined as early colonization and red marked times are defined as late colonization. **(B)** Skin explant viability by MTT assay. A reduction in color from yellow color to purple formazan has been observed in living skin biopsies for up to 8 days. **(C)** Relative *gyr*B mRNA expression over time. Exp, exponential phase; pxp, post-exponential phase.

Healthy human skin tissue was cultured at the air-liquid interface for up to 10 days (Figure 1A). To assess the viability of the skin explants we analyzed the ability of the tissue to reduce the MTT. Reduction from yellow color to purple formazan in living skin biopsies was observed for up to 8 days. At later time points, the cell viability decreased in skin explants (Figure 1B). In addition, we performed a validation of the integrity of our skin explants following the validation described by Neil et al. (2020). Using H&E stained slides, neither signs of spongiosis, necrosis or cell death could be observed. The epidermis and dermis are not separated over this period of time and no alterations in the corneal layer were observed. Furthermore, the terminal differentiation marker filaggrin and keratin 10 were unaffected during this period (Supplementary Figure 2). We decided to follow the *S. aureus* adaptation process for up to day 8. During this period, we confirmed stable colonization of the skin samples with *S. aureus* by demonstrating persistent expression levels of the house keeping gene *gyr*B (Figure 1C). *gyr*B transcripts were stable over time, both from skin samples that were colonized with exponentially grown bacteria as well as from skin samples colonized with post exponentially grown bacteria (mean *gyr*B exponential bacteria 2.20 × 10^4^ and mean *gyr*B post exponential bacteria 1.60 × 10^4^). As expected, *gyr*B values of bacteria grown *in vitro* (medium only without skin) increased over time, indicating a growth in the cell culture medium (Figure 1C).

### *S. aureus* Colonization of Human Skin Results in Uniform *agr* Inhibition Independent of the Inoculum Growth Status

**FIGURE 2:**
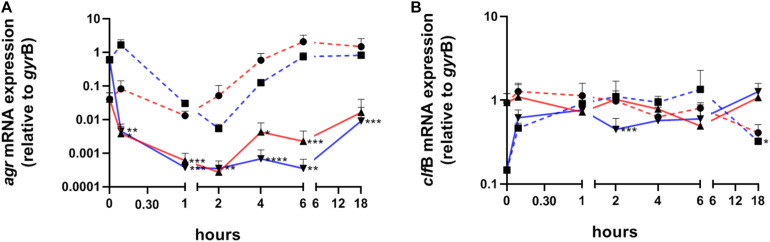
Transcriptional analysis of *S. aureus* colonizing human skin samples after exponential or post-exponential growth. Transcripts were quantified in reference to the transcription of *gyr*B in samples colonized with exponentially (red lines) or post-exponentially grown *S. aureus* (blue lines). Dashed lines represent growth *in vitro* (without skin and medium only). Values are the mean value of at least 3 colonized skin samples from different individuals. **(A)** Relative transcription of the accessory gene regulator (*agr*) and **(B)** of clumping factor B (*clf*B). Statistically significant differences between exponential or post-exponentially *ex vivo* samples and their corresponding *in vitro* samples were indicated. *P ≤ 0.05; **P ≤ 0.01; ***P ≤ 0.001; ****P ≤ 0.0001.

To profile the expression pattern of *S. aureus* during skin colonization we performed transcript analysis by quantitative real-time PCR (qPCR) starting 15 min after initial colonization. To evaluate, whether contact with the epidermis induced a uniform change in the expression pattern independently of the initial transcription level, we colonized skin samples with either exponentially or post-exponentially grown *S. aureus* (Figure 1A). In addition, wells containing medium only, were also inoculated with the corresponding inoculum to analyze gene expression during *in vitro* growth in the absence of skin contact. Initially, we analyzed transcription of RNAIII of the quorum sensing system *agr*, which is usually repressed during exponential growth, and transcription of *clf*B as marker for an exponentially expressed gene (Figure 2). Strikingly, *agr* transcription was immediately switched off upon bacterial contact to the skin surface. Notably, the massive *agr* downregulation also took place when skin samples were colonized with post-exponentially grown bacteria in which *agr* is highly active (Figure 2A). Thus, contact with skin led to a downregulation of *agr* in post-exponentially and exponentially grown bacteria and this downregulation lasted for up to 18 h. In contrast to *agr* suppression, transcription of *clf*B was induced in post exponentially grown bacteria upon skin contact or remained high when inoculated with expontential grown bacteria (Figure 2B). In summary, *S. aureus* contact with human skin results in consistent transcriptional changes independent of the inoculum’s growth phase.

### Comparative Analysis of Bacterial Gene Expression Between *ex vivo* Co-culture and *in vivo* Colonization of the Nasal Cavity

**FIGURE 3:**
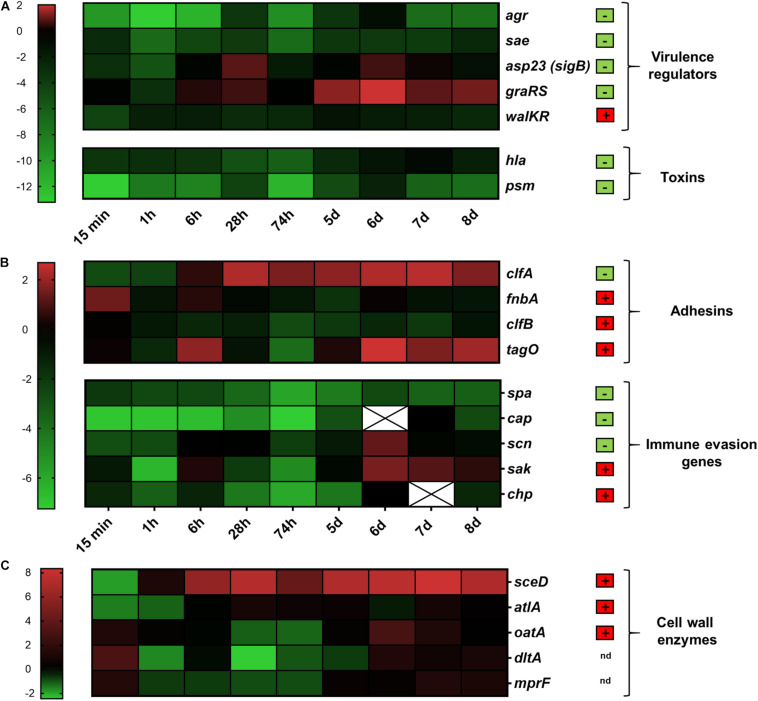
Transcriptional analysis during human skin colonization. Results are depicted as the ratio of transcription *ex vivo* vs. maximal expression *in vitro*. All data were log transformed (basis 2) and changes in gene expression were normalized in reference to the constitutively expressed gene *gyr*B. Genes colored red were up-regulated compared to *in vitro*; genes colored green were down-regulated compared to *in vitro*. Black indicates the same expression levels *ex vivo* and *in vitro*. White cells with an “x” have no values because gene expression was below the detection limit. Results are the mean values of 3 different skin samples from different individuals. **(A)** Genes belonging to virulence regulation and toxin production were clustered. **(B)** Genes involved in adhesion and immune evasion were combined. **(C)** Genes involved in cell wall modification. Genes relevant during nasal colonization were marked with a + (box with red background), while genes that were not relevant during nasal colonization were marked with a – (box with green background) (Burian et al., 2010b). Gene name abbreviations see Supplementary Table 2. The color chart was generated using GraphPad Prism 9.0.2.

Subsequently, we analyzed the expression of 21 *S. aureus* genes associated with a variety of cellular functions such as virulence regulation, toxin production, adhesion, immune evasion and cell wall dynamics. The expression of these genes during colonization of the human nasal cavity was previously assessed (Burian et al., 2010b) and was used for comparison (Figure 3). Skin explants were colonized with exponentially grown bacteria and gene expression was followed over 8 days during the colonization process. To define early and late colonization, time points up to 74 h were defined as early and 5–8 days as late colonization (Figure 1A). The results are summarized according to the functional categorization of target genes.

#### Global Regulators

In *S. aureus* a complex regulatory network is responsible for differential gene expression resulting in a characteristic *in vitro* pattern– high adhesion expression in the early stage of growth and high toxin production during the later stages (Cheung et al., 2004). We investigated the activity of five well-defined regulators: *agr*, *sae*, *sig*B (detected as the tightly *sig*B-dependent gene *asp*23), *gra*RS, and *wal*KR. The massive downregulation of *agr* described above persisted over the entire period of colonization (Figure 3A). Expression of the two-component systems *sae* and *wal*KR was not influenced by skin colonization (Figure 3A and Supplementary Figure 1A). In contrast, expression of *sig*B and *gra*RS was upregulated after contact with skin, especially during late colonization. Here, *gra*RS transcription even exceeded maximal transcription levels *in vitro*, suggesting a role of these two regulators during skin colonization (Figure 3A).

#### Toxins

Toxins such as the alpha-hemolysin (*hla*) or the phenol-soluble modulins (*psm*) are highly expressed during post-exponential growth as they are positively regulated by the *agr* system (Queck et al., 2008; Jenul and Horswill, 2019). In accordance to *agr* transcription, a massive downregulation was observed immediately after bacterial contact to the skin (Figure 3A).

#### Adhesins

*S. aureus* cell-wall-anchored proteins and other adhesive molecules are implicated in binding to host matrix molecules and may therefore be critical during the colonization process. Interestingly, the fibrinogen binding protein clumping factor A (*clf*A) and the fibrinogen and fibronectin binding protein *fnb*A, both *sig*B target genes (Goerke and Wolz, 2004), were strongly upregulated upon skin contact. Transcription of *clf*A remained constantly high over time and even exceeded the maximal transcription observed *in vitro* (Figure 3B and Supplementary Figure 1B).

*S. aureus* wall teichoic acid (WTA) is an important colonization factor mediating adherence to epithelial and endothelial cells (Weidenmaier and Peschel, 2008). We characterized the role of WTA during skin colonization by analyzing the transcription of the WTA biosynthesis gene *tag*O. In our study *tag*O transcription between day 6 and 8 constantly exceeded maximal *in vitro* transcription (Figure 3B).

#### Immune Evasion and Immune Modulatory Factors

Immune evasion is a prerequisite for stable and sustained colonization. In *S. aureus* it is mediated by a large number of molecules, such as protein A (s*pa*), the extracellular capsular polysaccharide (*cap*) and the prophage encoded immune evasion molecules staphylokinase (*sak*), staphylococcal complement inhibitor (*scn*) and chemotaxis inhibitory protein (*chp*). Transcript analysis of *spa* and *cap* revealed low expression levels whereas transcription of *scn* and *sak* was increased during late colonization (Figure 3B and Supplementary Figure 1B).

#### Cell-Wall Modification Enzymes

Enzymes involved in cell wall remodeling contribute to resistance against antimicrobial peptides and modulate the immune response. Expression of *oat*A, *dlt*A, and *mpr*F was only slightly altered upon skin contact. However, expression of *sce*D and *atl*A was highly induced throughout the course of skin colonization (Figure 3C).

In summary, the comparison between *ex vivo* skin co-culture and *in vivo* nose colonization revealed similar changes in gene expression for most genes as for example the strong inhibition of the *agr* system and the down regulation of genes encoding toxins or the high induction of *sce*D. However, the strong induction of *clf*A and *gra*RS seems to be specific for skin colonization.

### Proteases of All Three Catalytic Classes Were Strongly Transcribed Immediately After Contact of *S. aureus* With Human Skin

**FIGURE 4:**
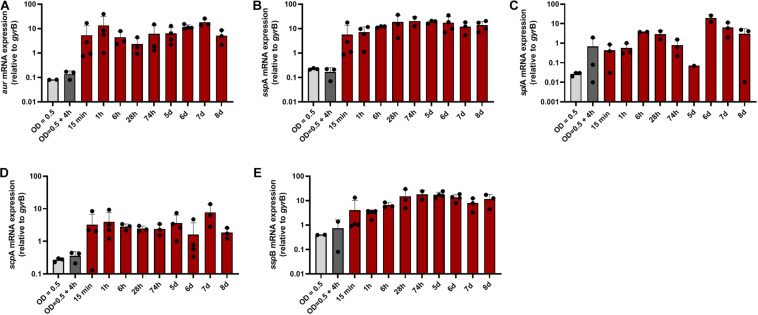
Transcription of protease-encoding genes during colonization of human skin. Transcripts were quantified in reference to the transcription of *gyr*B in skin samples colonized with *S. aureus*. **(A)** Relative transcription of the metalloprotease aureolysin (*aur*), the serine proteases *ssp*A (V8 protease) **(B)** and *spl*A **(C)**, and the cysteine proteases staphopain A and B [*scp*A **(D)** and *ssp*B **(E)**].

The data support that toxicity of *S. aureus* is dampened through inactivation of virulence regulators such as *agr*. Moreover, toxins might be inactivated through proteolytic cleavage (Gimza et al., 2021). On the other hand *S. aureus* secreted proteases might also contribute to tissue degradation and inactivation of host defense systems (Singh and Phukan, 2019). For *Staphylococcus epidermidis* the protease EcpA can be a deleterious component of the skin microbiome in AD (Cau et al., 2021). Therefore, we subsequently focused on the analysis of genes coding for major proteases: the metalloprotease aureolysin (*aur*), the serine proteases *ssp*A (V8 protease), and *spl*A (member of the protease-like proteins), and the cysteine proteases staphopain A and B (encoeded by *scp*A and *ssp*B). Transcription of all proteases was strongly induced during the entire time period of colonization starting already after 15 min with the exception of *spl*A, which was transcribed at low levels and peaked only during late colonization (Figure 4).

## Discussion

*S. aureus* is able to interact with its human host, both as a pathogen and as a commensal. The knowledge of bacterial gene regulation upon epidermal contact and the circumstances influencing gene regulation during skin colonization are crucial for understanding their role in infection. In the present study, we investigated *S. aureus* adaption to human skin using *ex vivo* skin explants. This model allowed us to longitudinally follow the colonization and the virulence factor expression in *S. aureus* as response to the contact with human skin tissue. Here, a differentiated expression pattern reflecting the adaptation to the epidermis was observed during early colonization. Notably, this response was independent of the growth phase of the *S. aureus* inoculum.

Most prominently, skin contact resulted in immediate inhibition of the quorum sensing system Agr. Of note, *agr* transcription was even lower than the expression in bacteria at the early exponential growth phase known to suppress the Agr system. Thus, the inhibition of *agr* expression can only in part be explained by culture dilution. Consistent with low *agr* transcription, typical *agr* target genes (*hla*, *cap, psm*) remained downregulated during skin exposure. Furthermore, the low *agr* transcription as well as the downregulation of *agr* target genes is consistent with the expression profile found during nasal colonization (Burian et al., 2010a,b). The strict shutdown of *agr* immediately upon contact to the skin indicates that at least during early colonization *agr* inactivation promotes bacterial colonization. On the native skin other staphylococcal species may further contribute to *agr* inhibition since some of them produce *agr* autoinducing molecules, which competitively inhibit the Agr system of *S. aureus* (Jenul and Horswill, 2019). Nevertheless, the global virulence regulator Agr and in particular the Agr dependent PSMs are known to significantly contribute to skin diseases such as AD (Williams et al., 2019) or abscess formation (Wright et al., 2005). One may speculate that Agr becomes activated once the bacteria breaches through the intact barrier of the skin. The mechanisms leading to this postulated Agr activation remain to be elucidated and will be the subject of future investigations.

In opposition to asymptomatic colonization of the nose, where WalKR seems to be one major regulator for adaptive gene expression (Burian et al., 2010b), *S. aureus* adaptation to the skin was correlated with activation of the antimicrobial peptide sensing system *gra*RS and the alternative sigma factor B. In accordance to *sig*B activity, expression of the *sig*B target genes *clf*A and *fnb*A (Goerke and Wolz, 2004) was also induced upon skin contact suggesting a role during adhesion. Thus, the gene expression profile during colonization of the skin differs partly from that during colonization of the human nose, especially in terms of global regulatory elements and in adhesion. Nevertheless, there are also remarkable similarities between skin and nasal colonization, for example the high transcription of the autolysins *sce*D and *atl*A (Burian et al., 2010b).

In addition to adhesion and cell wall remodeling, another essential step in colonization of the skin is the defense against the host’s immune response. In *S. aureus* immune evasion is mediated by a large number of molecules (Foster, 2005) including proteases. Besides their function in tissue degradation, interception of host enzymes and bacterial adhesion regulation (Dubin, 2003), proteases are able to inactivate antimicrobial peptides (Sieprawska-Lupa et al., 2004). For example, aureolysin and staphopain cleave and inactivate the human defensin peptide LL-37, which is involved in AD (Sieprawska-Lupa et al., 2004; Foster, 2005; Sonesson et al., 2017). Since both proteases were highly transcribed throughout the colonization process they might protect *S. aureus* from antimicrobial peptides and contribute to successful colonization. Interestingly, none of the skin colonizing bacteria can actively penetrate intact skin (Koziel and Potempa, 2013). Prerequisite for skin penetration is a defect of the epidermal integrity for example by small cuts. Therefore, it would be interesting to analyze the role of proteases during the switch from colonization to infection in an impaired skin barrier model, a possible scenario using our newly introduced *ex vivo* skin model (Marquardt et al., 2015).

## Conclusion and Outlook

By using our skin explant colonization model, we provide first insights into the adaptation strategy of *S. aureus* during the early phase of human skin colonization. We demonstrated that skin explants as well the inoculated *S. aureus* strain can survive over several days, providing a model to mimic asymptomatic carriage of *S. aureus* on healthy skin. The model will allow in the future to unravel the molecular mechanism of gene regulation under these specific conditions at the air-liquid interface and to obtain a more comprehensive picture of gene expression by for example RNAseq analysis. There are two major limitations in this study. First, the adaptation of *S. aureus* was evaluated neglecting the skin’s microbiota, which should be considered in future studies, and second, due to technical limitations, information on protein level could not be provided. From the preliminary data one can speculate that *S. aureus* is equipped with a yet not described “contact sensing system.” Moreover, the model should allow the analysis of bacterial interference over time and the impact of host immune factors such as cytokines on bacteria. Although both the nose and the epidermis are colonized by the microbe, there are considerable differences in regulatory elements and adhesion depending on the organ colonized. But the two organs also share the induction of bacterial pathways, such as the strong transcription of *sce*D. These common regulatory genes might be crucial for a successful decolonization strategy avoiding symptomatic skin infections. Virulence factors with significant expression in different body habitats might provide new targets for specific therapy and prevention.

## Data Availability Statement

The raw data supporting the conclusions of this article will be made available by the authors, without undue reservation.

## Ethics Statement

The studies involving human participants were reviewed and approved by the Ethics Commission at the Medical Faculty of RWTH University Aachen, Pauwelsstr. 30, 52074 Aachen, Germany. The patients/participants provided their written informed consent to participate in this study.

## Author Contributions

MB, CW, and AY performed the study concept and design. MB, AK, YM, and AY designed and performed the experiments. MB, JB, MH, CW, and AY wrote the manuscript. MB, JP, LS, AK, LH, CW, and AY analyzed the data. All authors contributed to the article and approved the submitted version.

## Conflict of Interest

The authors declare that the research was conducted in the absence of any commercial or financial relationships that could be construed as a potential conflict of interest.

## Publisher’s Note

All claims expressed in this article are solely those of the authors and do not necessarily represent those of their affiliated organizations, or those of the publisher, the editors and the reviewers. Any product that may be evaluated in this article, or claim that may be made by its manufacturer, is not guaranteed or endorsed by the publisher.
