# Supplementary material for: Adaptation of Staphylococcus aureus to the Human Skin Environment Identified Using an ex vivo Tissue Model
Source: Front Microbiol. 2021 Sep 21;12:728989. doi: 10.3389/fmicb.2021.728989 (PMC8490888; doi:10.3389/fmicb.2021.728989)
Supplement: Supplementary file 1 [file Data_Sheet_1.zip › Supplementary Figure 1.DOCX]

**Supplementary Figure 1**


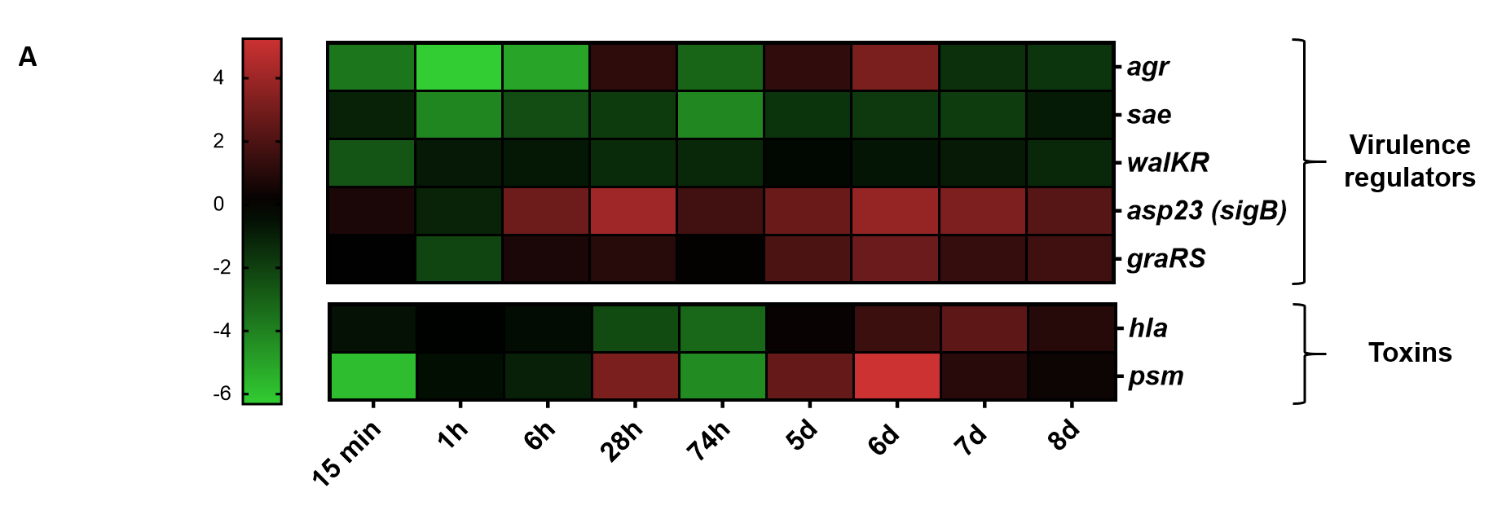


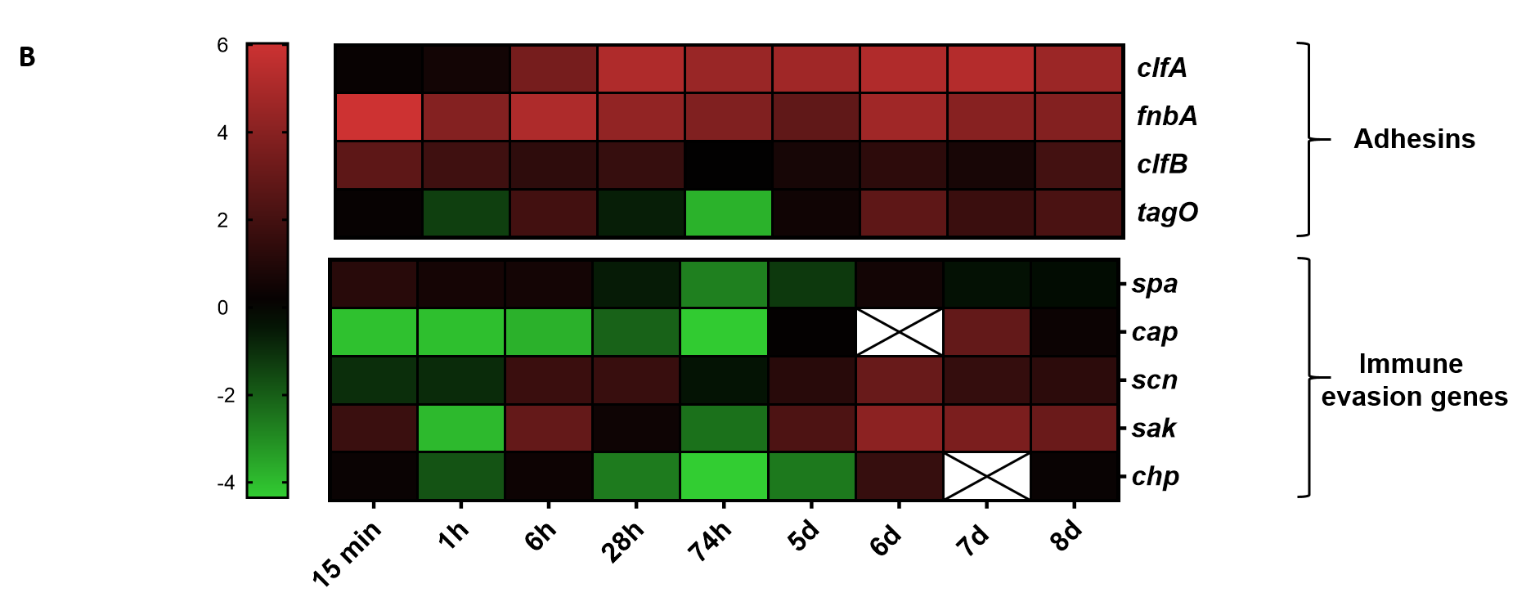


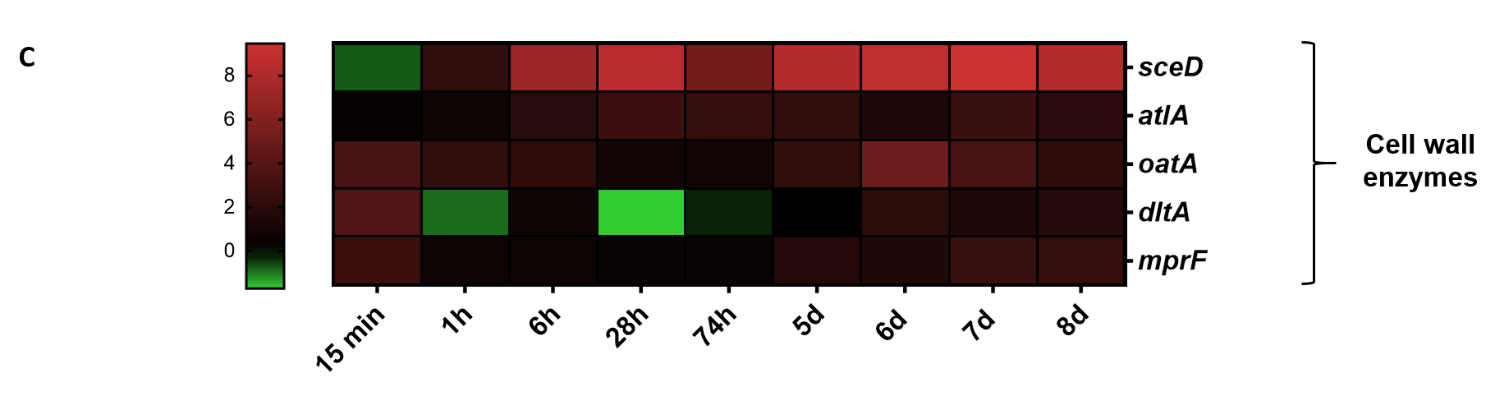


**Supplementary Figure 1. Transcriptional analysis during human skin colonization.** Results are depicted as the ratio of transcription *ex vivo* versus minimal expression *in vitro*. All data were log transformed (basis 2) and changes in gene expression were normalized in reference to the constitutively expressed gene *gyr*B. Genes colored red were up-regulated compared to *in vitro*; genes colored green were down-regulated compared to *in vitro*. Black indicates the same expression levels *ex vivo* and *in vitro*. White cells with an “x” have no values because gene expression was below the detection limit. Results are the mean values of 3 different skin samples from different individuals. (**A**) Genes belonging to virulence regulation and toxin production were clustered. (**B**) Genes involved in adhesion and immune evasion were combined. (**C**) Genes involved in cell wall modification. Gene name abbreviations see Supplementary Table 2. The color chart was generated using GraphPad Prism 9.0.2.
