# Supplementary material for: Adaptation of Staphylococcus aureus to the Human Skin Environment Identified Using an ex vivo Tissue Model
Source: Front Microbiol. 2021 Sep 21;12:728989. doi: 10.3389/fmicb.2021.728989 (PMC8490888; doi:10.3389/fmicb.2021.728989)
Supplement: Supplementary file 1 [file Data_Sheet_1.zip › Supplementary Figure 2.DOCX]

**Supplementary Figure 2**

**A**


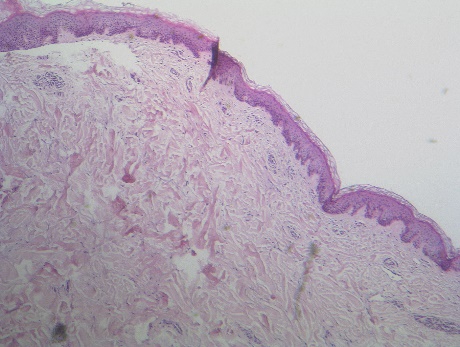

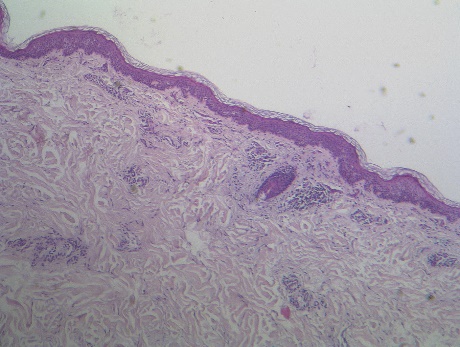

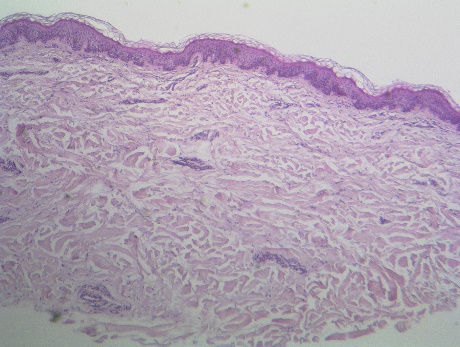
**Day 1 Day 2 Day 3**


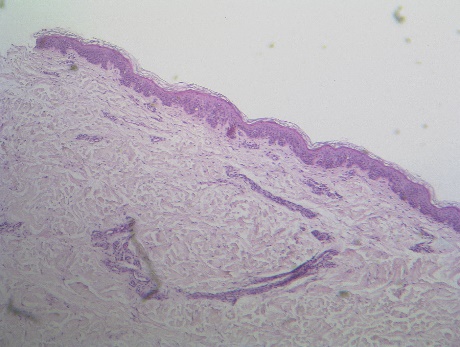

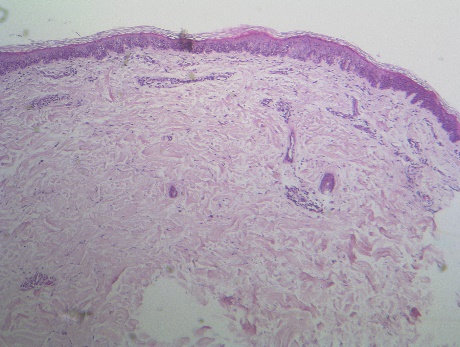

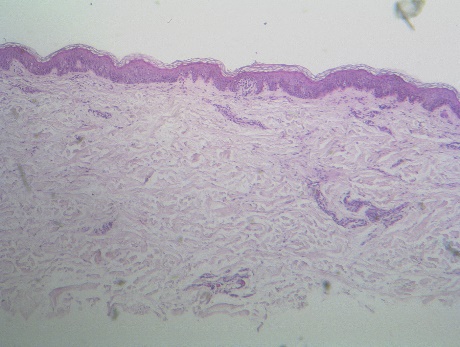
**Day 4 Day 5 Day 6**


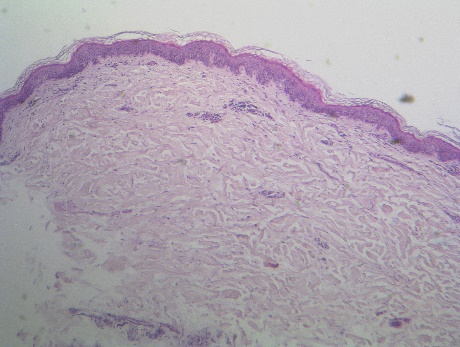

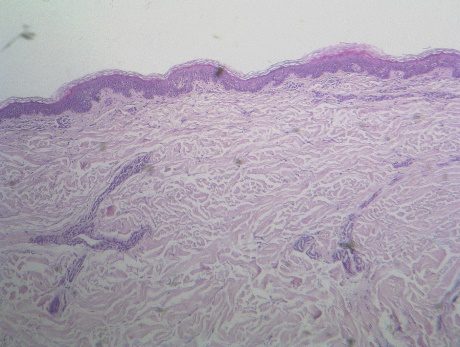

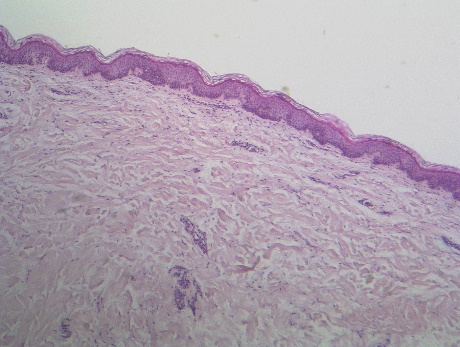
**Day 7 Day 8 Day 9**

**B**

**
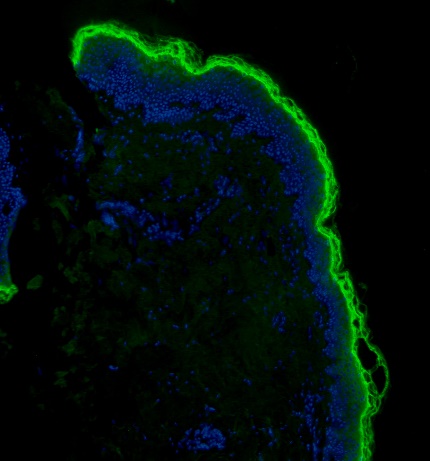

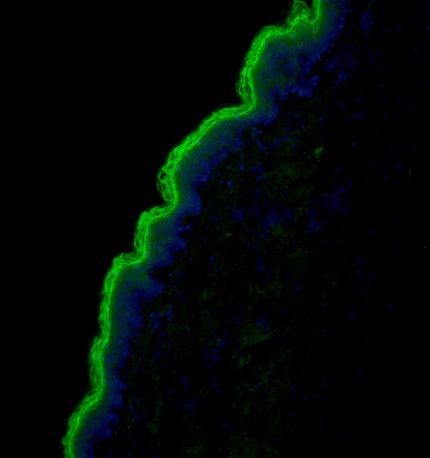

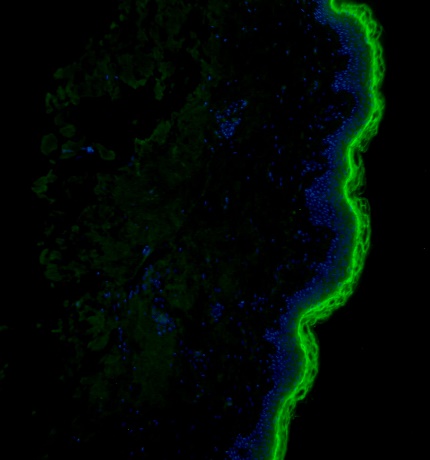
Day 1 Day 2 Day 3**


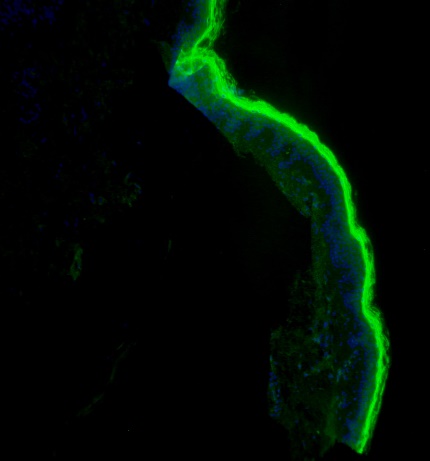
**
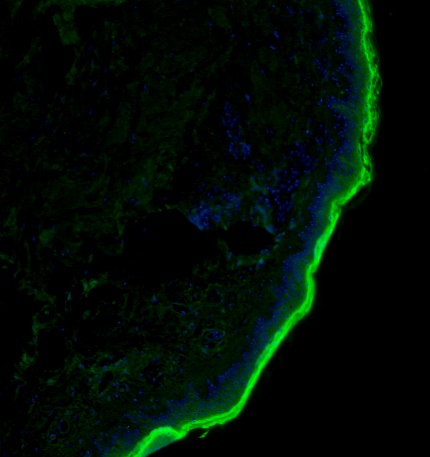

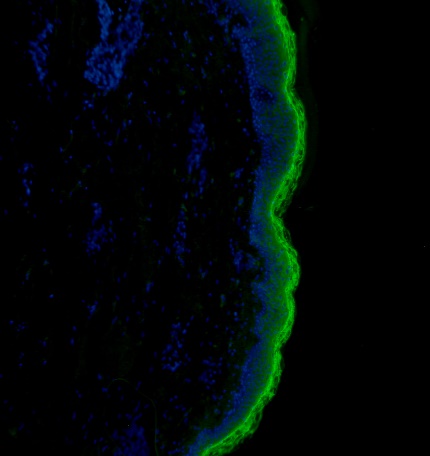
Day 4 Day 5 Day 6**


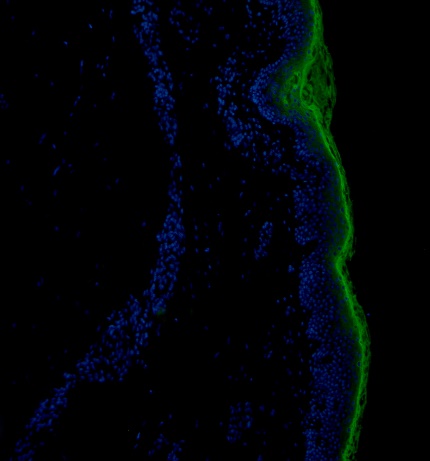

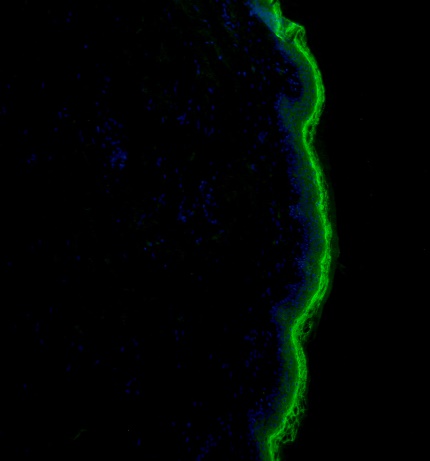

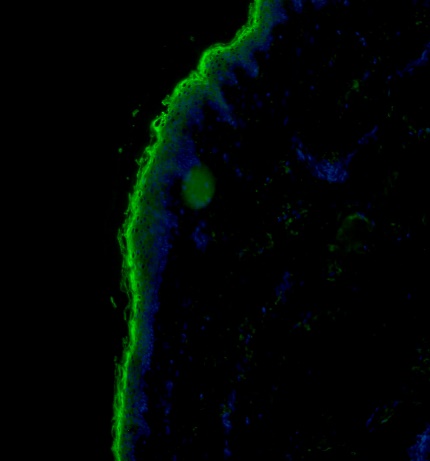
**Day 7 Day 8 Day 9**

**C**

**
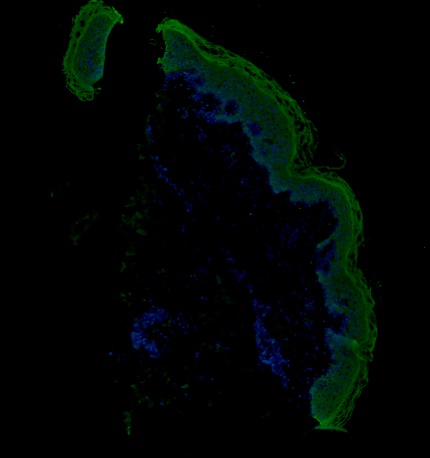

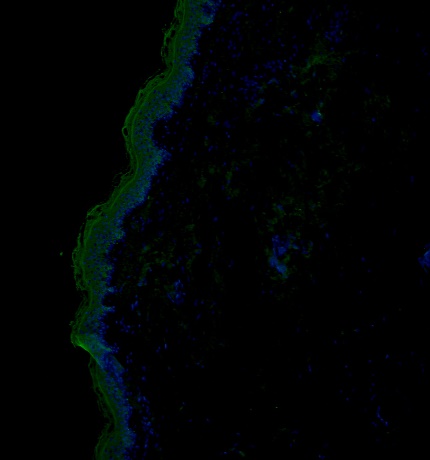

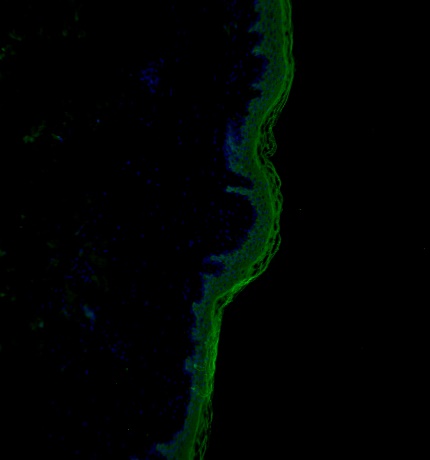
Day 1 Day 2 Day 3**

**
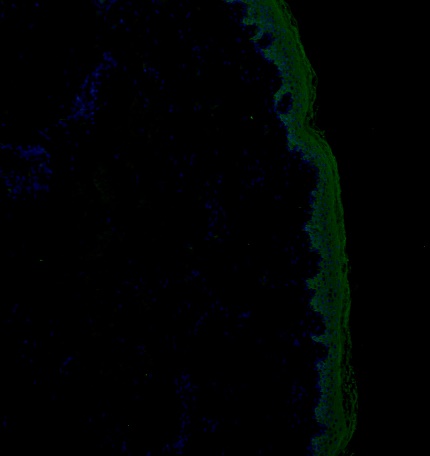

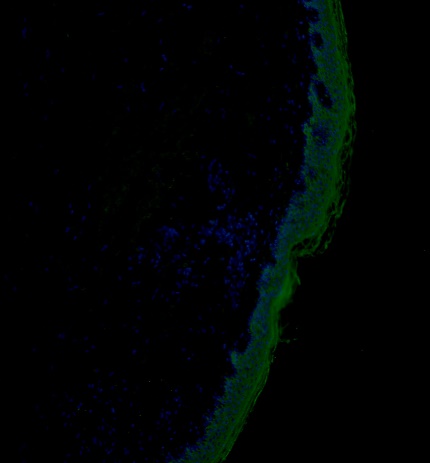

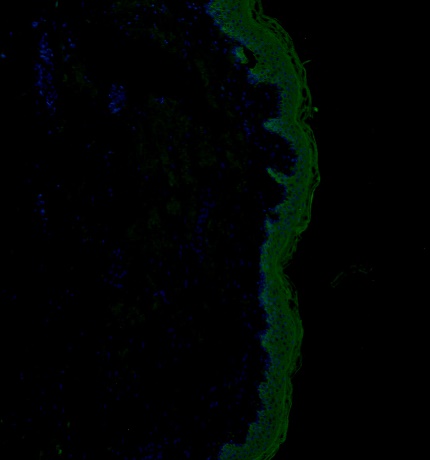
Day 4 Day 5 Day 6**


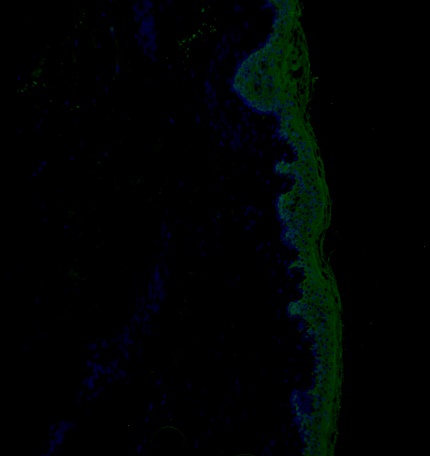

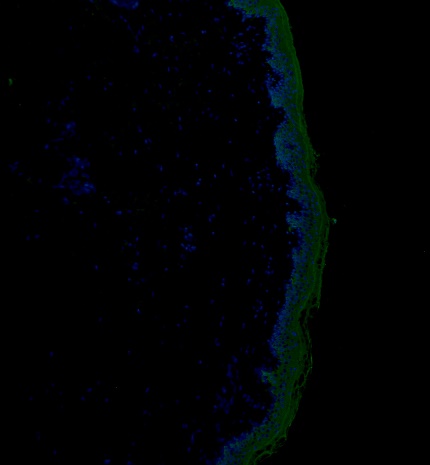

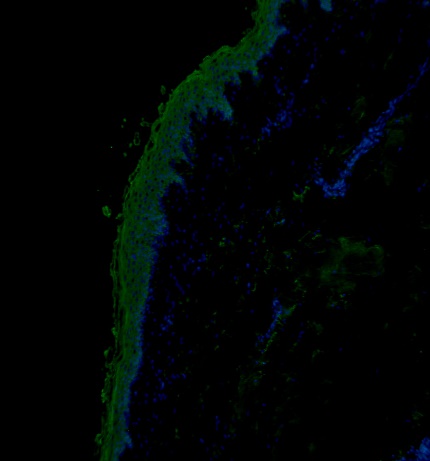
**Day 7 Day 8 Day 9**

**D**

**
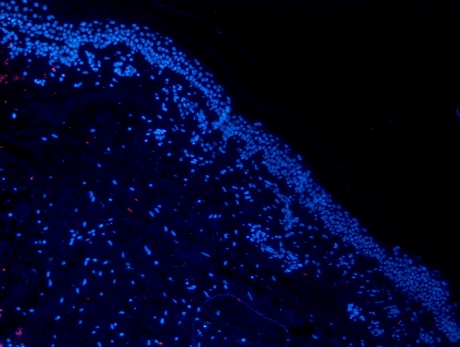

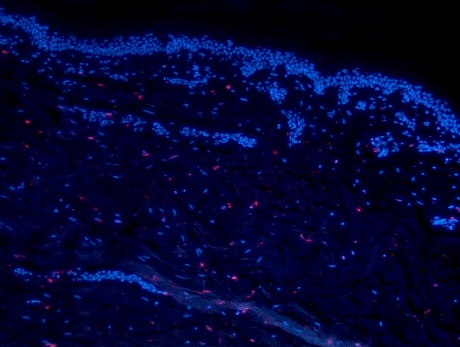

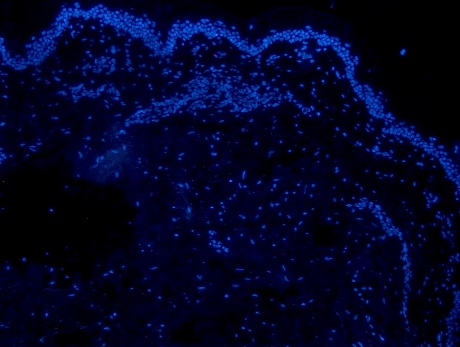
Day 1 Day 2 Day 3**

**
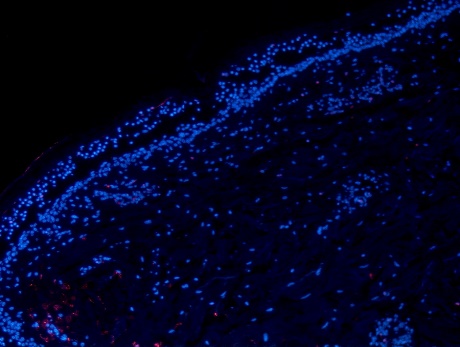

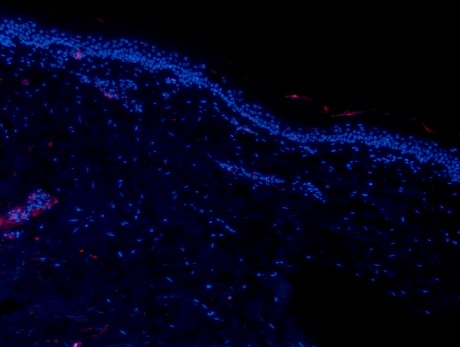

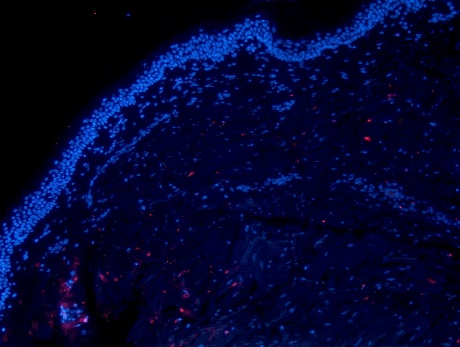
Day 4 Day 5 Day 6**


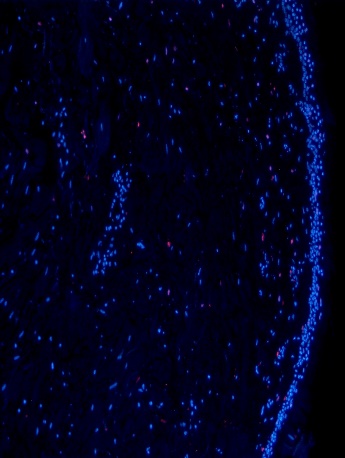


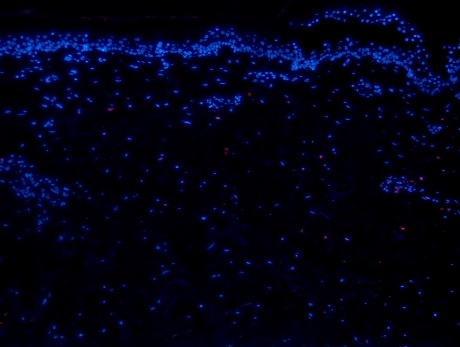
**
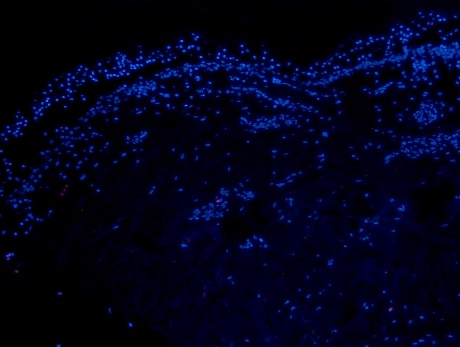
Day 7 Day 8 Day 9**

**E F**

**G**

**Supplementary Figure 2. Tissue integrity over time.** Haematoxylin and eosin staining of skin tissue over 9 days (**A**). Immunofluorescence of filaggrin (**B**) and keratin 10 (**C**) and TUNEL staining (**D**). Quantification of fluorescence intensity was measured at three representative positions per image for filaggrin (**E**) and keratin 10 (**F**). Quantification of cell death in the epidermal layer was measured at 10 representative positions per skin explant (**G**). Representative images from one donor are illustrated.
