# Supplementary material for: Adaptation of Staphylococcus aureus to the Human Skin Environment Identified Using an ex vivo Tissue Model
Source: Front Microbiol. 2021 Sep 21;12:728989. doi: 10.3389/fmicb.2021.728989 (PMC8490888; doi:10.3389/fmicb.2021.728989)
Supplement: Supplementary file 1 [file Data_Sheet_1.zip › Supplementary Table 1.DOCX]

Supplementary Table 1: **Oligonucleotide primers**

| **Target gene** | **Primer** | **Primer sequence** | | **Purpose** | |  |
| --- | --- | --- | --- | --- | --- | --- |
| *aur* | aur_L_A2 | | GGAAATTTTCAAGATATGCATTTACA | | qPCR | |
|  | aur R A2 | | GCTGCTGGTGATAAAGTGCTC | | qPCR | |
| *ssp*A | sspA_L | | GACAACAGCGACACTTGTGAG | | qPCR | |
|  | sspA R | | GTGGATGATTGTCCATAGCTTTT | | qPCR | |
| *ssp*B | sspB_L | | CCTAATCAAATGATTGAATACGGTAA | | qPCR | |
|  | sspB_R | | GATGGTACGCCTTCTTGATAATG | | qPCR | |
| *scp*A | scpA L | | TTTTAGAGATGTCAAAAGCAGGAAT | | qPCR | |
|  | scpA_R | | TCCAAAATGGTAGTTCACTCCTC | | qPCR | |
| *spl*A | splA L | | GCGGGTGGTACTGGTGTAGT | | qPCR | |
|  | splA_R | | CGCAAGGTCTTCTTTTCCAG | | qPCR | |
