# Supplementary material for: Adaptation of Staphylococcus aureus to the Human Skin Environment Identified Using an ex vivo Tissue Model
Source: Front Microbiol. 2021 Sep 21;12:728989. doi: 10.3389/fmicb.2021.728989 (PMC8490888; doi:10.3389/fmicb.2021.728989)
Supplement: Supplementary file 1 [file Data_Sheet_1.zip › Supplementary Table 2.DOCX]

Supplementary Table 2: ***S. aureus* genes analyzed by qPCR**

| **Product** | **Gene** | **Function** |
| --- | --- | --- |
| **Virulence regulators** |  |  |
| Accessory gene regulator | *agr* | Virulence regulator |
| *S. aureus* exoprotein-expression | *sae* | Virulence regulator |
| Regulator of cell wall metabolism and virulence | *wal*KR | Virulence regulator |
| Alkaline shock protein | *asp23* (*sig*B) | Strictly *sig*B regulated gene |
| Antimicrobial peptide-sensing system | *gra*RS | Virulence regulator |
| **Toxins** |  |  |
| α-Hemolysin | *hla* | Pore forming hemolysin |
| Phenol-soluble modulin | *psm* | Cytolytic peptides |
| **Adhesins** |  |  |
| Clumping factor A | *clf*A | Adhesion |
| Clumping factor B | *clf*B | Adhesion |
| Fibronectin binding protein A | *fnb*A | Adhesion |
| Teichoic acid glycerol | *tag*O | WTA biosynthesis |
| **Cell wall and related enzymes** |  |  |
| *Staphylococcus carnosus* exoprotein D | *sce*D | Lytic transglycosylase |
| Major autolysin | *atl*A | Bifunctional peptidoglycan hydrolase |
| O-acetyltransferase A | *oat*A | O-acetylation of peptidoglycan |
| D-alanine-D-alanyl carrier protein ligase | *dlt*A | D-alanine modification of teichoic acids |
| Multiple peptide resistance factor | *mpr*F | Modification of membrane lipids with L-Lysine |
|  |  |  |
| **Immune evasion genes** |  |  |
| Immunoglobulin G binding protein A | *spa* | IgG binding/Immune evasion |
| Capsular polysaccharide serotype 5 and 8 | *cap*A | Anti-phagocytosis |
| Staphylokinase | *sak* | Immune modulation (phage encoded) |
| Chemotaxis inhibitory protein | *chp* | Immune modulation (phage encoded) |
| Staphylococcal complement inhibitor | *scn* | Immune modulation (phage encoded) |
| **Proteases** |  |  |
| Aureolysin | *aur* | Metallo-protease |
| Serine protease (V8 protease) | *ssp*A | Serine protease |
| Staphopain B | *ssp*B | Cysteine protease |
| Staphopain A | *scp*A | Cysteine protease |
| Serine Protease like protein A | *spl*A | Serine protease |
